# Prophage Induction and Differential RecA and UmuDAb Transcriptome Regulation in the DNA Damage Responses of *Acinetobacter baumannii* and *Acinetobacter baylyi*

**DOI:** 10.1371/journal.pone.0093861

**Published:** 2014-04-07

**Authors:** Janelle M. Hare, Joshua C. Ferrell, Travis A. Witkowski, Alison N. Grice

**Affiliations:** Department of Biology and Chemistry, Morehead State University, Morehead, Kentucky, United States of America; The University of Sydney, Australia

## Abstract

The SOS response to DNA damage that induces up to 10% of the prokaryotic genome requires RecA action to relieve LexA transcriptional repression. In *Acinetobacter* species, which lack LexA, the error-prone polymerase accessory UmuDAb is instead required for *ddrR* induction after DNA damage, suggesting it might be a LexA analog. RNA-Seq experiments defined the DNA damage transcriptome (mitomycin C-induced) of wild type, *recA* and *umuDAb* mutant strains of both *A. baylyi* ADP1 and *A. baumannii* ATCC 17978. Of the typical SOS response genes, few were differentially regulated in these species; many were repressed or absent. A striking 38.4% of all ADP1 genes, and 11.4% of all 17978 genes, were repressed under these conditions. In *A. baylyi* ADP1, 66 genes (2.0% of the genome), including a CRISPR/Cas system, were DNA damage-induced, and belonged to four regulons defined by differential use of *recA* and *umuDAb*. In *A. baumannii* ATCC 17978, however, induction of 99% of the 152 mitomycin C-induced genes depended on *recA*, and only 28 of these genes required *umuDAb* for their induction. 90% of the induced *A. baumannii* genes were clustered in three prophage regions, and bacteriophage particles were observed after mitomycin C treatment. These prophages encoded *esvI*, *esvK1*, and *esvK2*, ethanol-stimulated virulence genes previously identified in a *Caenorhabditis elegans* model, as well as error-prone polymerase alleles. The induction of all 17978 error-prone polymerase alleles, whether prophage-encoded or not, was *recA* dependent, but only these DNA polymerase V-related genes were de-repressed in the *umuDAb* mutant in the absence of DNA damage. These results suggest that both species possess a robust and complex DNA damage response involving both *recA-*dependent and *recA*-independent regulons, and further demonstrates that although *umuDAb* has a specialized role in repressing error-prone polymerases, additional regulators likely participate in these species' transcriptional response to DNA damage.

## Introduction

Cells that experience damage to their DNA have evolved mechanisms of sensing, repairing, and replicating this damaged DNA. In most bacteria, DNA damage from various sources such as UV radiation, alkylating chemicals (e.g. mitomycin C (MMC)), and antibiotics can induce up to 10% of the genome in this SOS response [1]. Induced SOS genes encode proteins that sense damage, control cell division, and repair, replicate and recombine DNA for continued cellular survival [2]–[4]. These processes are often carried out in an error-free manner, using conserved SOS genes such as *ssb*, *recA*, *recN, ruvA, ruvB, uvrA, uvrB*, and *uvrD* in repair and recombination processes [3] and *sulA* in controlling the bacterial cell cycle [5], [6]. However, DNA damage left unrepaired can also lead to the induction of SOS gene products that carry out error-prone replication of this damaged DNA. These error-prone polymerases, formed by the homodimerization of UmuC and two molecules of self-cleaving UmuD (DNA polymerase V, [7]), or DinB/DinP (DNA polymerase IV [8]) are responsible for SOS mutagenesis.

The mechanism by which these SOS genes are specifically transcribed when the cell experiences DNA damage is through relief of LexA repression [9]. This de-repression occurs after RecA binds ssDNA, an indicator of DNA damage [10], and induces LexA self-cleavage [11]. The normal state of repression in the absence of DNA damage thus prevents constitutive production of the entire SOS regulon, and SOS mutagenesis.

This general model of SOS gene induction and function, which has been developed to a significant extent in *Escherichia coli* [2], [4], is conserved throughout proteobacterial classes, albeit imperfectly. Gammaproteobacteria in the order *Enterobacteriales* often possess one LexA protein that recognizes a conserved SOS box in SOS gene promoters [2]. However, in the *Pseudomonadales* (containing the opportunistic, often multidrug-resistant pathogens *Acinetobacter baumannii* and *Pseudomonas aeruginosa*) and *Xanthomonadales* orders, more divergent responses to DNA damage exist. For example, *Pseudomonas putida* possesses two different LexA proteins, each controlling separate regulons [12], and *Geobacter sulfurreducens* also has two LexA proteins, which do not bind the *recA* promoter [13]. This diversity highlights the need for additional examination of not only the mechanisms of SOS gene control but also SOS gene identity in this order.

Further components of the SOS model of gene regulation are absent in *Acinetobacter* species of this order. None of its fellow members of the family *Moraxellaceae* possess *umuD* homologs [14], which may have implications for the ability of these organisms to undergo SOS mutagenesis after DNA damage. Additionally, no *lexA* homolog has been identified in this genus [14]. Nevertheless, in the non-pathogenic genetic model organism *Acinetobacter baylyi* ADP1 [15], previous investigations of the DNA damage response demonstrated that two genes are induced by mitomycin C and UV exposure in this strain. These two induced genes are *recA* (which unlike in other bacteria, does not require *recA* for its own induction [16], nor contains an SOS box in its promoter [16]), and *ddrR*, a gene of unknown function found only in the genus *Acinetobacter* [14]. *ddrR* is transcribed divergently from *umuDAb* [17], which is itself an unusual component of the DNA damage response of this species. UmuDAb is a UmuD homolog that is required for full induction of *ddrR* [17], but it is not known whether ADP1 uses it to induce other genes that are, in other bacteria, part of the SOS response. UmuDAb carries out self-cleavage in a RecA-dependent manner after cells experience diverse forms of DNA damage, and thus shares features with both the DNA polymerase V component UmuD and the LexA repressor [18]. Recent work demonstrates that in *A. baumannii* ATCC 17978, UmuDAb binds to and represses the promoters of *umuDC* homologs [19] and so might serve as a LexA analog for this genus.

Multiple *umuD* and *umuC* homologs co-exist in *A. baumannii* strains, and at least some of these strains (ATCC 17978, AB0057) display DNA damage-induced mutagenesis [14]. These observations suggest that these strains possess a mechanism of sensing DNA damage and inducing at least error-prone polymerase production under these conditions, and suggest that specialized UmuD function has evolved in this species. Whether this mechanism is a global response to DNA damage, induces SOS genes found in other species, or requires the action of RecA and/or other repressors is unknown.

These unusual features in the DNA damage responses of *Acinetobacter* species prompted us to use RNAseq experiments to define the transcriptome of *A. baylyi* ADP1 after DNA damage, and compare its response to that of the opportunistic pathogen, *A. baumannii* ATCC 17978. Our aims in these experiments were to determine both the existence and identity of any global DNA damage-induced transcriptome in these species, and the possible requirements for RecA and UmuDAb in regulating such a response. Although UmuDAb has been shown to regulate some DNA damage-induced genes [17], [19], the limited similarity between *umuDAb* and *lexA* [14] suggests that it may not directly substitute for all LexA function, and allows for the possibility that additional regulators might exist. This stress response of this pathogen is also relevant, as environmental stresses such as dessication and exposure to UV radiation used for decontamination [20] are encountered in health care settings where nosocomial *Acinetobacter* pathogens abound. Examination of DNA damage and stress responses have been specifically identified as areas in which our knowledge of *Acinetobacter* virulence is lacking [21].

We observed that the organization, gene content, and regulation of the induced and repressed genes in the mitomycin C-induced DNA damage transcriptome differed significantly between *A. baylyi* ADP1 and *A. baumannii* ATCC 17978. These experiments also established different uses for RecA in these two species' DNA damage responses, and suggested that UmuDAb is only one of multiple repressors of the DNA damage response in both species, serving a specialized role in regulating the transcription of error prone polymerases throughout the genome. These error-prone polymerase genes, as well as known virulence-associated genes, were found in bacteriophage particles in *A. baumannii* ATCC 17978 after DNA damage, which could facilitate the spread of mutation-inducing and other virulence genes to other bacteria.

## Materials and Methods

### Bacterial strains and growth conditions

*A. baylyi* strains ADP1, ACIAD1385 (Δ*recA*::KanR), and ACIAD2729 (Δ*umuDAb*::KanR) [22] as well as *A. baumannii* strains ATCC 17978, its isogenic *recA* insertion mutant [23], and a Δ*umuDAb*::KanR null mutant were grown at 37°C in minimal media plus succinate [17] for transcriptome and RT-qPCR analyses, and in Luria-Bertani broth for the production of phage particles. For both RNASeq transcriptome and RT-qPCR analyses, a 3 ml overnight culture, grown at 37°C at 250 rpm, was diluted 1:25 into 5 ml fresh media and grown with shaking for two hours, at which time the culture was split in two and 2 μg/mL mitomycin C (MMC) was added to one culture. Further incubation for three hours served to induce gene expression. DNA damage-induced mutagenesis after UV-C exposure was conducted as described previously [14].

### Mutant strain construction

Null mutations of *umuDAb* (A1S_1389), *umuD* (A1S_0636) and *rumB* (A1S_1173) in *A. baumannii* ATCC 17978 were constructed by replacing the coding sequence of each gene with either the kanamycin resistance gene from the Invitrogen pCRII vector (for *umuDAb* and *umuD*), or the streptomycin/spectinomycin resistance cassette from pUI1638 (for *rumB*), as described previously [22]. Primer sequences are listed in Table S1; “up” primers amplified DNA upstream of each coding sequence, and “dw” primers amplified DNA downstream of each coding sequence. The kanamycin resistance gene was amplified with primers Kmup and Kmdw [24] and the streptomycin/spectinomycin resistance cassette was amplified from pUI1638 [25] with primers StrepSpecFor and StrepSpecRev. Splicing overlap extension PCR was used to construct linear DNA fragments from these three pieces, 300 ng of which was electroporated into *A. baumannii* ATCC 17978 cells. (In the *rumB* replacement, the linear fragment was first cloned into the suicide vector pEX18Gm before electroporation [26]). Transformants were selected on LB plates containing 30 μg/mL kanamycin or 10 μg/mL each of streptomycin and spectinomycin. Mutants were confirmed with PCR analyses to contain allelic replacements of the wild type allele and were not merodiploids.

### RNASeq experiments and analyses

RNA was purified from one milliliter samples (biological triplicates for 17978; duplicates for ADP1) and processed through the Epicentre MasterPure RNA Purification kit. Further removal of contaminating DNA was performed using the Ambion DNA-*free* rigorous DNase treatment. RNASeq experiments were conducted with the assistance of Cofactor Genomics (St. Louis). RNA quality was assessed on a BioRad Experion instrument to have a quality corresponding to an RNA Integrity Number equal or greater than 9. Whole transcriptome RNA was extracted from total RNA by removing large and small ribosomal RNA (rRNA) using RiboMinus Bacterial Kit (Invitrogen). Five ug of total RNA was hybridized to rRNA-specific biotin-labeled probes at 70°C for 5 minutes. The rRNA-probe complexes were then removed by streptavidin-coated magnetic beads, and rRNA free transcriptome RNA was concentrated using ethanol precipitation.

In cDNA synthesis, 1 μg of transcriptome RNA was incubated with fragmentation buffer (Illumina RNA-seq kit) for 5 minutes at 94°C. Fragmented RNA was purified with ethanol precipitation. First-strand cDNA was prepared by priming the fragmented RNA using random hexamers and followed by reverse transcription using Superscript II (Invitrogen). The second-strand of cDNA was synthesized by incubation with second-stranded buffer, RNase Out and dNTP (Illumina RNA-seq kit) on ice for 5 minutes. The reaction mix was then treated with DNA Pol I and RNase H (Invitrogen) at 16°C for 2.5 hours.

In constructing cDNA libraries, double-stranded cDNA was treated with a mix of T4 DNA polymerase, Klenow large fragment and T4 polynucleotide kinase to create blunt-ended DNA, to which a single 3′ A base was added using Klenow fragment (3′ to 5′ exo-) provided by an Illumina RNA-seq kit. Size selection of adaptor-ligated DNA was performed by cutting the target fragment out of a 4–12% acrylamide gel. The amplified DNA library was obtained by in-gel PCR using a Phusion High-Fidelity system (New England Biolabs).

Sequencing and cluster generation was performed according to the sequencing and cluster generation manuals from Illumina (Cluster Station User Guide and Genome Analyzer Operations Guide). Primary data were generated using the Illumina Pipeline version SCS 2.8.0 paired with OLB 1.8.0. NovoAlign version 2.07.05 was used for all sequence alignment; aligner algorithm specifics can be obtained from novocraft.com. The coverage depth for sequencing of the *A. baylyi* ADP1 libraries was an average of 67.5-fold for the wild type strain, 69.7-fold for ACIAD1385, and 148-fold for ACIAD2729, with an average percent coverage of reference bases of 95.8%, 98.2%, and 99.8%, respectively. Coverage depth for the sequenced *A. baumannii* ATCC 17978 libraries was 235-fold for the wild type strain (99.3% of reference bases covered), 238-fold for the *recA* strain (98.7% of reference based covered), and 203-fold for the *umuDAb* strain (97.3% of reference bases covered). An average of 878 million to 1.1 billion total bases were generated for each *A. baumannii* ATCC 17978 library and over 300 million total bases were generated for each *A. baylyi* ADP1 library. Clusters were linearly normalized by multiplying each sample's coverage by the total reads of the lower read-count sample divided by the respective sample's total reads, and the induction ratio of reads between MMC-treated and untreated samples was then calculated. Genes were considered induced if this ratio was greater than or equal to 2.0, and considered repressed if this ratio was less than or equal to 0.5 and if the expression levels in at least two wild type uninduced samples were above the detection threshold. The detection threshold for each sample corresponded to one Illumina read in a million that aligned to the reference genome sequences (CP000521, CP000522, and CP000523 for *A. baumannii* ATCC 17978 [27] and its two plasmids, respectively; CR543861.1 for *A. baylyi* ADP1 [28]). These sequence datasets have been submitted to the NCBI Short Read Archive (http://www.ncbi.nlm.nih.gov/sra) under accession number SRP036862.

### RT-qPCR analysis

RNA samples for RT-qPCR were produced from 1 mL of triplicate biological samples with the Epicentre MasterPure RNA Purification Kit, after which additional removal of DNA was performed using Ambion DNA-*free* rigorous DNaseI treatment. Removal of contaminating DNA was verified by the absence of PCR products amplified when PCR was performed with primers listed in Table S2 and S3: 17978umuDCRTFor and 17978umuDCRTRev for *A. baumannii* strains 17978, 17978 *recA*, and 17978 Δ*umuDAb*. For *A. baylyi* strains ADP1 and ACIAD1385, umuDAb#2RTRev and umuDAb#RTFor were used, and for ACIAD2729, dnaNRTFor and dnaNRTRev were used. Genomic DNA was used as a positive control. RNA sample quality was confirmed on an E-Gel EX 2% agarose gel (Invitrogen) before use.

cDNA was synthesized from 1 μg of RNA with oligo(dT) and random hexamers by a modified Moloney murine leukemia virus reverse transcriptase in a 25 μL reaction (Bio-Rad iScript cDNA Synthesis kit). Five μL of a 1:100 dilution of this cDNA was used to perform RT-qPCR using BioRad iTaq SYBR Green Supermix on an Applied Biosystems 7300 Real-Time PCR system in a 15 μL reaction. Technical triplicates were run for each of the three biological replicates in ABI MicroAmp Optical 96-well clear reaction plates, with the following cycling conditions: 95°C for 5 minutes, followed by 40 cycles of 95°C for 15 seconds and 60°C for 1 minute. A dissociation step of 95°C for 15 seconds, followed by 60°C for 30 seconds and 95°C for 15 seconds was used to check product integrity. No template controls for each primer set confirmed absence of product formation. Each RT-qPCR plate contained wild type and one mutant strain sample, comparing reference primers and test primers for the gene of interest.

RT-qPCR primers were designed using PrimerBlast (NCBI) and are listed in Tables S2 and S3. PCR efficiency was evaluated for every primer set by dilution of genomic DNA over 5 logs of template concentration and was between 94% and 105% for all primer sets. Efficiency was calculated using the formula E = 10^(−1/slope)^ of the standard curve generated with the primer set, where efficiency = (E-1)×100%. Primer concentration used was 400 μM. All test gene primer sets were compared to the reference gene primer set 16SrRNA#RTFor and 16SrRNA#2RTRev for *A. baylyi* ADP1 (Table S2) and 1797816rRNARTFor and 1797816rRNARTRev for *A. baumannii* ATCC 17978 (Table S3). Validation of these reference primers was performed by observing no significant difference between MMC-treated and untreated samples in either *A. baumannii* ATCC 17978 or *A. baylyi* ADP1 in six independent experiments (p>0.05 in a paired t-test). Transcriptional changes were calculated using the 2^−ΔΔCT^ method [29] and GraphPad InStat was used to conduct all statistical analyses.

### Bacteriophage purification, electron microscopy and analyses

Phages were produced in cultures grown in LB broth. Overnight cultures were diluted 1:25 into fresh medium and grown at 37°C with shaking for 1 hour before MMC at 2 μg/mL was added. The cultures' optical density at 600 nm was measured each hour for six additional hours after induction with MMC. At either three or six hours, 1 mL of culture was centifuged at 13,000 rpm for two minutes, and the supernatant was filtered through a 0.22 μm filter. This filtrate was centrifuged at 13,200 rpm for one hour at room temperature and the pellet was resuspended in phage buffer (10 mM Tris, pH 7.5, 10 mM MgSO_4_, 68.5 mM NaCl, 1 mM CaCl_2_). These samples were processed through the Ambion DNA-free DNase Treatment & Removal kit if PCR analyses were performed.

The resulting phage suspension was processed for transmission electron microscopy. Phage samples were placed on freshly made carbon coated formvar grids for 5 minutes, rinsed with phage buffer and deionized water for ten seconds each, and stained twice with 1% uranyl acetate for one minute each. Uranyl acetate was wicked off and the grid was air dried. Micrographs were taken using 80kV accelerating voltage on a JEOL 100CX transmission electron microscopy onto Kodak 4489 film, then scanned with a Minolta Dimage Scan Multi Pro film scanner at 2400 dpi. The capsid diameter, tail width, and tail length of twelve phage particles observed in micrographs was measured with Image J software [30]. The arithmetic mean of the measurements was reported. Analysis of the genome structure and content of the three cryptic prophage regions was performed using the web server Phage Search Tool (PHAST) [31].

## Results

Previous reports had indicated that *ddrR* [17] and *recA* [16] were induced by DNA damage in *A. baylyi* ADP1, and recent observations indicated that multiple error prone polymerases were induced by various forms of DNA damage in *A. baumannii* ATCC 17978 [19], [32]. However, in the absence of a LexA homolog encoded by these species [14], it was not known whether multiple genes were induced in these species, nor how this response might be regulated. RNA-Seq experiments were performed to test whether *A. baylyi* ADP1 and *A.baumannii* ATCC 17978 (henceforth abbreviated as ADP1 and 17978, respectively) possessed a genome-wide transcriptional response to mitomycin C exposure. Genes were considered induced (or repressed) if their expression increased (or decreased) by 2.0-fold or more, relative to their expression in untreated cultures.

### *A. baylyi* ADP1 possess a DNA damage transcriptomes of SOS response genes, a CRISPR/Cas system, and other genes

Sixty-six genes, or 2.0%, of all ADP1 genes were induced (Table 1), indicating a global system of regulating gene expression in response to this form of DNA damage. These 66 induced genes were widely dispersed throughout the chromosome, and included 8 putative operons of two genes each. In addition to these induced genes, an astonishing 38.4% of all ADP1 genes were repressed upon DNA damage.

**Table 1:** Genes induced in *A. baylyi* ADP1 after MMC-induced DNA damage and their regulation by UmuDAb and RecA.

Gene identity	Gene name	Function	Regulation	Fold Induction
ACIAD1385	*recA*	DNA recombination and repair	NA*	5.2
ACIAD0445	*gst*	Glutathione *S*-transferase (detoxification)	Neither	190.0
ACIAD2480		Conserved hypothetical protein	Neither	8.5
ACIAD2482	*csy3*	RAMP superfamily protein/Cas system	Neither	8.5
ACIAD2483	*cas6*	Endoribonuclease involoved in crRNA biogenesis	Neither	8.3
ACIAD0446		Conserved hypothetical protein	Neither	7.9
ACIAD3449	*ssb*	RecBCD nuclease ssDNA-binding protein	Neither	7.4
ACIAD0724	*nrdA*	Ribonucleoside diphosphate reductase, alpha subunit	Neither	7.0
ACIAD2481	*csy2*	RAMP superfamily protein/Cas system	Neither	6.5
ACIAD0722	*nrdB*	Ribonucleoside-diphosphate reductase, beta subunit	Neither	5.4
ACIAD0005		Conserved hypothetical protein	Neither	5.3
ACIAD3565		Conserved hypothetical protein	Neither	4.6
ACIAD3649		Conserved hypothetical protein	Neither	3.9
ACIAD2210	*rpmE*	50S ribosomal protein L31	Neither	3.6
ACIAD3535	*raiA*	Stress response ribosomal inhibitor	Neither	3.0
ACIAD1205	*dps*	Stress response DNA-binding protein, starvation induced resistance to H_2_O_2_, ferritin-like	Neither	2.8
ACIAD2479		Conserved hypothetical protein	Neither	2.8
ACIAD3545		Putative esterase	Neither	2.5
ACIAD2295		Putative oxidoreductase	Neither	2.5
ACIAD3566		Hypothetical protein	Neither	2.5
ACIAD2652	*gyrA*	DNA gyrase, subunit A, type II topoisomerase	Neither	2.4
ACIAD0151	*guaA*	Glutamine aminotransferase	Neither	2.3
ACIAD3390		Putative acetyl-CoA hydrolase/transferase	Neither	2.3
ACIAD3503	*guaB*	IMP dehydrogenase	Neither	2.2
ACIAD0010		Putative Fe/S cluster chaperone	Neither	2.2
ACIAD0868		Conserved hypothetical protein	Neither	2.1
ACIAD1473		Conserved hypothetical protein	*recA*	5.2
ACIAD2484	*cas1*	DNAse	*recA*	3.7
ACIAD1772		Conserved hypothetical protein	*recA*	3.7
ACIAD3455	*uvrA*	Excinuclease ABC subunit A	*recA*	3.4
ACIAD2614	*ruvA*	Holliday junction helicase subunit A	*recA*	2.4
ACIAD0002	*dnaN*	DNA polymerase III, beta chain	*recA*	2.2
ACIAD2613	*dgt*	dGTPase	*recA*	2.1
ACIAD3408		Endonuclease G	*recA*	2.1
ACIAD2729	*umuDAb*	Component of DNA polymerase V	*recA* **	4.0
ACIAD2730	*ddrR*	DNA damage-inducible protein	*recA* and *umuDAb*	26.2
ACIAD1478	*hemO*	Heme oxygenase	*recA* and *umuDAb*	3.2
ACIAD1474		Conserved hypothetical protein	*recA* and *umuDAb*	2.5
ACIAD0334	*fadA*	3-ketoacyl-CoA thiolase	*recA* and *umuDAb*	2.4
ACIAD0335	*fadB*	Fatty oxidation complex alpha subunit	*recA* and *umuDAb*	2.3
ACIAD2034		Putative signal peptide protein	*recA* and *umuDAb*	2.2
ACIAD0387	*acuA*	Fimbrial-like protein	*recA* and *umuDAb*	2.2
ACIAD1208		Fatty acid desaturase	*recA* and *umuDAb*	2.1
ACIAD2103	*ahpC*	alkyl hydroperoxide reductase (detoxification)	*recA* and *umuDAb*	2.1
ACIAD1024		Conserved hypothetical protein	*recA* and *umuDAb*	2.1
ACIAD0697		*ompA*-like	*recA* and *umuDAb*	2.0
ACIAD0006		Hypothetical protein	*umuDAb*	4.2
ACIAD0401	*rpsO*	30S ribosomal protein S15	*umuDAb*	2.6
ACIAD3325	*rpoZ*	RNA polymerase, omega subunit	*umuDAb*	2.3
ACIAD3602		Conserved hypothetical protein	*umuDAb*	2.3
ACIAD1402	*iscA*	Iron-binding protein	*umuDAb*	2.3
ACIAD3506	*aceF*	Dihydrolipoamide *S*-acetyltransferase	*umuDAb*	2.2
ACIAD3309		Lipase	*umuDAb*	2.2
ACIAD0316	*htpG*	Heat shock protein; chaperone Hsp90	*umuDAb*	2.1
ACIAD3654	*dnaK*	Heat shock protein; chaperone Hsp70	*umuDAb*	2.1
ACIAD0728		Conserved hypothetical protein	*umuDAb*	2.1
ACIAD3564		Conserved hypothetical protein	*umuDAb*	2.1
ACIAD3155	*mdh*	Malate dehydrogenase	*umuDAb*	2.0
ACIAD0042		Acyl carrier	*umuDAb*	2.0
ACIAD3604		Putative histidine triad	*umuDAb*	2.0
ACIAD2938	*rpmA*	50S ribosomal protein L27	*umuDAb*	2.0
ACIAD3330	*bfrB*	Bacterioferritin	*umuDAb*	2.0
ACIAD3370		Conserved hypothetical protein	*umuDAb*	2.0

*recA expression could not be evaluated in the null *recA* mutant, but was not regulated by *umuDAb*.

** *umuDAb* expression could not be evaluated in the null *umuDAb* mutant, but was regulated by *recA*.

A core set of 6 SOS genes for gamma proteobacteria such as *Acinetobacter* includes *recA*, *ssb*, *ruvA*, *ruvB*, *recN*, and *uvrA* [33], with a larger set of 36 genes induced in *Escherichia coli* [2] and *Pseudomonas aeruginosa* [34], the best-studied organism in the order to which *Acinetobacter* belongs (*Pseudomonadales*). Surprisingly, only 6 of these 36 genes were induced, and 7 genes were repressed, while 9 genes were neither induced nor repressed (Table 2). ∼40% of all SOS genes (*dinI*, *dinG*, *hokE*, *lexA*, *molR*, *pcsA*, *polB*, *sbmC*, *sulA*, *ybfE*, *ydjM*, *ydjQ*, *yebG*, *yigN*; all of which are present in *E. coli* and some of which are present in *P. aeruginosa*) are not encoded in the ADP1 genome, although none of these were ‘core’ SOS genes. RT-qPCR experiments confirmed that *recA, ssb*, *umuDAb*, and *ddrR* were each induced >2-fold.

**Table 2:** Regulation and presence of canonical SOS genes in *Acinetobacter* species.

Gene	*A. baylyi* ADP1	*A. baumannii* 17978
*recA*	Induced	Induced
*ssb*	Induced	Induced
*umuDAb*	Induced	Induced
*dnaN*	Induced	No Change
*ruvA*	Induced	No Change
*uvrA*	Induced	No Change
*umuD* (0636*)	Not in genome	Induced
*umuC* (0637)	Not in genome	Induced
*umuD* (1174)	Not in genome	Induced
*rumB* (1173)	Not in genome	Induced
*umuC* (2015)	Not in genome	Induced
*holB*	Repressed	Repressed
*ruvC*	Repressed	Repressed
*uvrC*	Repressed	No Change
*dinB/dinP*	Repressed	No Change
*recN*	Repressed	No Change
*recG*	Repressed	No Change
*dnaQ*	Repressed	No Change
*ftsK, gyrB, hupB, polA, recF, ruvB, uvrB, uvrD,*	No Change	No Change
*dinI, dinG, hokE, lexA, molR, pcsA, polB, sbmC, sulA, ybfE, ydjM,ydjQ, yebG, yigN*	Not encoded in genome	Not encoded in genome

* Four digit numbers correspond to A1S gene numbers in *A. baumannii*.

In addition to the SOS response genes involved in recombination and repair, the induced genes included nucleotide metabolism-related *nrdAB* (ACIAD0722/0724) encoding ribonucleotide reductase, *dgt* (ACIAD2613), involved in breakdown of ssDNA (a trigger of the SOS response, [10]), and endonuclease G (ACIAD3408) (Table 1). Numerous genes encoded chaperones (*htpG*, ACIAD0316; *acuD/papD*, ACIAD0388) or other proteins involved in oxidative or other stresses, notably *dnaK* (ACIAD3654), *hemO* (heme oxygenase, ACIAD1478), *dps* (ACIAD1205), and the ribosomal inhibitor *raiA* (ACIAD3535). Detoxification enzymes encoded by *aphC* (ACIAD2103) and *gst* (ACIAD0445) were also induced. *gst* encodes a member of the glutathione *S*-transferase family, which is involved in oxidative stress and xenobiotic/antimicrobial agent detoxification [35] and was the most highly induced gene in both species. Additionally, *acuA* (ACIAD0387), which encodes the thin pilus subunit mediating ADP1 adhesion [36] was induced, as were the adaptive immunity Cascade proteins associated with a Type I-F CRISPR/Cas locus (ACIAD2479-2484) identified by the CRISPRFinder web server [37]. ACIAD2481 and 2482 were further confirmed to be induced with RT-qPCR experiments (data not shown).

**Figure 1:**
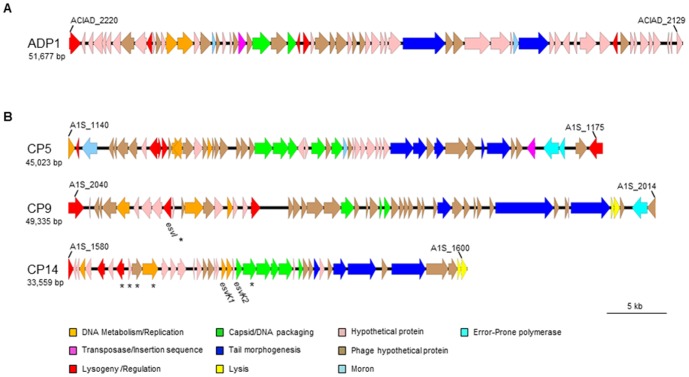
Comparison of the ADP1 and 17978 chromosomal regions indicated as putative prophages. This to-scale diagram indicates the size and predicted gene functions from PHAST analysis within the chromosomal regions designated as putative prophages. Locations in the genome are represented by the ACIAD and A1S gene designation boundaries for ADP1 and 17978, respectively. (**A**) All genes represented in the ADP1 prophage region were not induced after DNA damage, and approximately one-third of the genes in this region were either hypothetical conserved phage genes, or genes of predicted phage function (with only 2 each of DNA metabolism/replication, capsid/packaging and tail genes). (**B**) Three chromosomal regions of induced genes in *A. baumannii* ATCC 17978 overlapped with three regions of the genome designated as putative prophages by our PHAST analysis [31] and as cryptic prophages CP5, CP9 and CP14 [38]. Genes that were not induced in this study are marked with asterisks. Previously described virulence-associated *esv* genes [27] are named below each gene, and error-prone polymerase alleles are shown in bright cyan color (see legend). The specific gene function of each gene in order of its placement in each prophage is described in Table 4.

No genes were induced that are involved in natural competence, or were in either of the two putative prophage regions identified for ADP1 [28]. It is not known whether these putative prophages are functional [28]. One of these two prophages regions may be too small (9 kb) to encode a functional prophage, while the other, although possessing an appropriate genome size (53 kb), seems not to encode several proteins (e.g. in DNA replication and capsid and tail structural elements) normally required to produce functional bacteriophage particles, and only one third of its genes are of phage origin (Figure 1A).

### The DNA damage transcriptome of *A. baumannii* ATCC 17978 includes three prophages

In *A. baumannii* ATCC 17978, 152 genes, or 3.7% of the genome, was induced (Table 3), indicating that the expression of multiple genes was regulated in response to this form of DNA damage. In 17978, as for ADP1, few of the canonical SOS genes responded to MMC-induced DNA damage as in the *E. coli* model (Table 2). Only 4 of these 36 genes were induced, with core SOS genes *recA*, *umuD* and *ssb* induced, similar to ADP1. Two genes were repressed (*holB* and *ruvC*, also repressed in ADP1), while 16 genes were neither induced nor repressed, and the same 14 genes as were absent from the ADP1 genome were absent in the 17978 genome. There was no significant difference between ADP1 and 17978 in the number of genes in these four classes (induced, repressed, unaffected, absent) as tested with Chi-square analysis (p>0.05).

**Table 3:** Genes induced in *A. baumannii* 17978 after MMC-induced DNA damage and their regulation by UmuDAb and RecA.

Gene identity	Gene name	Function	Regulation	Fold Induction
A1S_1962	*recA*	DNA strand exchange and recombination*	Neither	5.6
A1S_0408	*gst*	Glutathione *S*-transferase* (detoxification)	*recA*	73.5
A1S_2026		Hypothetical protein	*recA*	30.8
A1S_3617		Hypothetical protein	*recA*	23.0
A1S_3764		Hypothetical protein	*recA*	20.4
A1S_3618		Hypothetical protein	*recA*	18.0
A1S_3779		Hypothetical protein	*recA*	17.3
A1S_1159		Hypothetical protein	*recA*	17.0
A1S_3765		Hypothetical protein	*recA*	16.8
A1S_3762		Hypothetical protein	*recA*	15.2
A1S_2027		Hypothetical protein	*recA*	14.7
A1S_1145		Putative Cro protein	*recA*	14.5
A1S_1157		Hypothetical protein	*recA*	14.2
A1S_2021		Hypothetical protein	*recA*	13.6
A1S_1156		Hypothetical protein	*recA*	13.5
A1S_2024		Glutamate 5-kinase	*recA*	13.4
A1S_1161		Hypothetical protein	*recA*	12.6
A1S_1158		Putative signal peptide	*recA*	12.5
A1S_1162		Hypothetical protein	*recA*	12.0
A1S_1163		Hypothetical protein	*recA*	11.7
A1S_3614		Hypothetical protein	*recA*	11.4
A1S_1160		Hypothetical protein	*recA*	11.3
A1S_2022		Putative tail fiber	*recA*	10.9
A1S_2031		Hypothetical protein	*recA*	10.8
A1S_3608		Hypothetical protein	*recA*	10.7
A1S_3615		Hypothetical protein	*recA*	10.5
A1S_3766		Hypothetical protein	*recA*	10.0
A1S_3763		Hypothetical protein	*recA*	9.8
A1S_3760		Hypothetical protein	*recA*	9.2
A1S_3772		Hypothetical protein	*recA*	9.2
A1S_2018		Tail tape measure protein	*recA*	9.2
A1S_2029		Hypothetical protein	*recA*	9.2
A1S_3611		Hypothetical protein	*recA*	9.1
A1S_1147		DNA methylase	*recA*	9.1
A1S_3761		Hypothetical protein	*recA*	9.1
A1S_1155		Putative phage-related protein	*recA*	8.9
A1S_3613		Hypothetical protein	*recA*	8.9
A1S_3754		Hypothetical protein	*recA*	8.9
A1S_1146		Site-specific methylase	*recA*	8.8
A1S_3606		Hypothetical protein	*recA*	8.7
A1S_3607		Hypothetical protein	*recA*	8.7
A1S_3758		Hypothetical protein	*recA*	8.7
A1S_3700		Hypothetical protein	*recA*	8.6
A1S_3757		Hypothetical protein	*recA*	8.5
A1S_2023		Hypothetical protein	*recA*	8.5
A1S_1166		Hypothetical protein	*recA*	8.4
A1S_3612		Hypothetical protein	*recA*	8.3
A1S_1149		Hypothetical protein	*recA*	8.2
A1S_2019		Hypothetical protein	*recA*	8.1
A1S_3771		Hypothetical protein	*recA*	8.0
A1S_2016		Phage-related lysozyme	*recA*	8.0
A1S_3616		Hypothetical protein	*recA*	7.9
A1S_3609		Hypothetical protein	*recA*	7.8
A1S_3776		Hypothetical protein	*recA*	7.8
A1S_3773		Hypothetical protein	*recA*	7.8
A1S_1153		Putative phage-related protein	*recA*	7.7
A1S_1595		Hypothetical protein	*recA*	7.7
A1S_2017		Hypothetical protein	*recA*	7.7
A1S_3767		Hypothetical protein	*recA*	7.6
A1S_1148		Hypothetical protein	*recA*	7.5
A1S_2036		DNA cytosine methyltransferase	*recA*	7.5
A1S_1152		Putative helicase	*recA*	7.3
A1S_1154		Putative bacteriophage protein	*recA*	7.3
A1S_2033		Hypothetical protein	*recA*	7.3
A1S_1167		Hypothetical protein	*recA*	7.1
A1S_1169		Hypothetical protein	*recA*	7.1
A1S_2039		Phage nucleotide binding protein	*recA*	7.1
A1S_1586	*esvK1*	Ethanol-stimulated virulence protein**	*recA*	7.1
A1S_1594		Hypothetical protein	*recA*	7.1
A1S_1164		Putative phage tail tape measure protein	*recA*	7.0
A1S_3620		Hypothetical protein	*recA*	7.0
A1S_3755		Holin	*recA*	7.0
A1S_1151		Hypothetical protein	*recA*	6.7
A1S_1165		Putative phage tail tape measure protein	*recA*	6.7
A1S_3605		Hypothetical protein	*recA*	6.7
A1S_2030		Putative phage associated protein	*recA*	6.7
A1S_1150		Hypothetical protein	*recA*	6.6
A1S_1168		Hypothetical protein	*recA*	6.5
A1S_3610		Hypothetical protein	*recA*	6.2
A1S_3778		Hypothetical protein	*recA*	6.2
A1S_3756		Hypothetical protein	*recA*	6.1
A1S_2032		Hypothetical protein	*recA*	6.0
A1S_1171		Hypothetical protein	*recA*	5.8
A1S_3777		Hypothetical protein	*recA*	5.8
A1S_3768		Hypothetical protein	*recA*	5.8
A1S_3678		Hypothetical protein*	*recA*	5.8
A1S_1170		Hypothetical protein	*recA*	5.7
A1S_3603		Hypothetical protein*	*recA*	5.7
A1S_3604		Hypothetical protein	*recA*	5.7
A1S_3621		Hypothetical protein	*recA*	5.7
A1S_3770		Hypothetical protein	*recA*	5.6
A1S_1591		Major capsid protein	*recA*	5.6
A1S_3696		Hypothetical protein	*recA*	5.6
A1S_3702		Hypothetical protein	*recA*	5.3
A1S_2035		Hypothetical protein	*recA*	5.3
A1S_3699		Hypothetical protein	*recA*	5.1
A1S_3769		Hypothetical protein	*recA*	5.0
A1S_3693		Hypothetical protein	*recA*	4.9
A1S_3287	*ssb*	RecBCD nuclease ssDNA-binding protein*	*recA*	4.9
A1S_1143		Hypothetical protein	*recA*	4.7
A1S_3705		Hypothetical protein	*recA*	4.7
A1S_1175		Phage integrase	*recA*	4.6
A1S_3759		Hypothetical protein	*recA*	4.4
A1S_3695		Hypothetical protein	*recA*	4.4
A1S_1172		Putative transposase	*recA*	4.3
A1S_3694		Hypothetical protein	*recA*	4.3
A1S_2038		Hypothetical protein	*recA*	4.2
A1S_1592		Putative Phage head-tail adaptor	*recA*	4.0
A1S_3703		Hypothetical protein	*recA*	4.0
A1S_1599		Hypothetical protein	*recA*	3.7
A1S_3685		Hypothetical protein*	*recA*	3.6
A1S_1593		Hypothetical protein	*recA*	3.5
A1S_1597		Phage tail tape measure protein	*recA*	3.5
A1S_1596		Hypothetical protein	*recA*	3.1
A1S_1598		Hypothetical protein	*recA*	3.1
A1S_3697		Hypothetical protein	*recA*	3.1
A1S_3701		Hypothetical protein	*recA*	3.0
A1S_1587	*esvK2*	Terminase; Ethanol-stimulated virulence protein**	*recA*	2.9
A1S_1589		Hypothetical protein	*recA*	2.9
A1S_1590		Peptidase U35 phage prohead HK97	*recA*	2.9
A1S_3804		Hypothetical protein*	*recA*	2.9
A1S_1600		Lysozyme	*recA*	2.6
A1S_3698		Hypothetical protein	*recA*	2.6
A1S_3727		Hypothetical protein*	*recA*	2.1
A1S_2025		Hypothetical protein	*recA* and *umuDAb*	19.1
A1S_1388	*ddrR*	DNA damage-inducible protein*	*recA* and *umuDAb*	13.7
A1S_2028		Phage putative head morphogenesis protein	*recA* and *umuDAb*	10.1
A1S_1174	*umuD*	DNA polymerase V component	*recA* and *umuDAb*	9.6
A1S_0636	*umuD*	DNA polymerase V component*	*recA* and *umuDAb*	8.0
A1S_3767		Hypothetical protein	*recA* and *umuDAb*	7.6
A1S_1173	*rumB*	DNA-directed DNA polymerase	*recA* and *umuDAb*	5.8
A1S_3619		Hypothetical protein	*recA* and *umuDAb*	5.4
A1S_3622		Hypothetical protein	*recA* and *umuDAb*	5.1
A1S_1144		Repressor, S24 family peptidase	*recA* and *umuDAb*	4.9
A1S_3759		Hypothetical protein	*recA* and *umuDAb*	4.4
A1S_2015	*umuC*	DNA-directed DNA polymerase	*recA* and *umuDAb*	3.8
A1S_1389	*umuDAb*	DNA polymerase V component*	*recA* and *umuDAb*	3.8
A1S_3704		Hypothetical protein	*recA* and *umuDAb*	3.3
A1S_1598		Hypothetical protein	*recA* and *umuDAb*	3.1
A1S_2040		Putative phage integrase	*recA* and *umuDAb*	3.0
A1S_3701		Hypothetical protein	*recA* and *umuDAb*	3.0
A1S_0637	*umuC*	DNA-directed DNA polymerase*	*recA* and *umuDAb*	2.8
A1S_1600		Lysozyme	*recA* and *umuDAb*	2.6
A1S_0278	*trnT*	Threonine tRNA*	*recA* and *umuDAb*	2.5
A1S_2014		SOS response associated thiol autopeptidase	*recA* and *umuDAb*	2.2
A1S_3774		Hypothetical protein	*recA* and *umuDAb*	2.2
A1S_3775		Hypothetical protein	*recA* and *umuDAb*	2.2
A1S_2236	*trnW*	Tryptophan tRNA*	*recA* and *umuDAb*	2.2
A1S_2034		Hypothetical protein	*recA* and *umuDAb*	2.1
A1S_3706		Hypothetical protein	*recA* and *umuDAb*	2.1
A1S_0421		Protein chain initiation factor IF-1*	*recA* and *umuDAb*	2.1
A1S_2020		Hypothetical protein	*umuDAb*	7.0

*Indicates that the induced gene is not part of a prophage region.

** See reference [27].

In contrast to the dispersal of induced genes throughout the chromosome in ADP1, the location of 90% of all 17978 induced genes nearly perfectly overlapped with three regions predicted to contain prophages by our analysis with the Phage Search Tool (PHAST), a web server designed to rapidly and accurately identify, analyze, and annotate prophages [31] (Figure 1B). These three regions were also the only regions to be identified as cryptic prophages (CP; CP5, CP9, and CP14) in 17978, based on their presence in some but not all epidemic-associated *A. baumannii* strains [38]. Ninety-nine percent of all genes within these prophage regions were induced.

These cryptic prophages encode some of the error-prone polymerase components that were induced in 17978: *umuDrumB* (A1S_1173-1174) in CP5, and A1S_2014-2015 in CP9. (A1S_2014, putatively transcribed in an operon with A1S_2015, belongs to a newly described family of SOS response associated thiol autopeptidases (SRAP; [39]) and was therefore included in the category of error-prone polymerase components.) Non-phage associated polymerases or polymerase components included *umuDAb* (A1S_1389) and a *umuDC* operon (A1S_0636-0637) that is located in a genomic island that may have been horizontally acquired from a *Yersinia* plasmid [38]. Notably, all error prone polymerases or polymerase components, as well as the conserved *ddrR* gene adjacent to *umuDAb*, were induced in both the RNASeq and additional RT-qPCR experiments. Both *umuD* and *umuC* genes in each operon were induced, although the level of induction in each case was greater for *umuD* than *umuC*, consistent with its position at the beginning of the operon and with its use in a 2:1 ratio to the *umuC* gene product in DNA polymerase V activity [7].

Virulence-associated genes were also induced in 17978. Previous studies in a *Caenorhabditis elegans* model of *A. baumannii* ATCC 17978 infection demonstrated that ethanol-stimulation of virulence was dependent upon 12 *esv* genes [27]. Two of these, *esvK1* and *esvK2*, which were encoded in CP14, were induced, with the induction of *esvK1* further tested and confirmed in RT-qPCR experiments. While the induction of *esvI*, encoded by CP9, fell just below the RNASeq induced cutoff ratio of 2.0, it was induced ∼5-fold after MMC treatment in RT-qPCR experiments. No CRISPR-Cas system is present in 17978, although some isolates of the EU clone lineage I possess a CRISPR-Cas system [40].

Although none of the genes on the 17978 plasmids pAB1 and pAB2 were induced, 6 of 11 pAB1 genes, and 2 of 6 pAB2 genes were repressed. Overall, 11.4% of all 17978 genes were repressed.

### *The A. baylyi* DNA damage transcriptome includes four different regulons of MMC-induced genes

In the SOS response of gammaproteobacteria, RecA action is typically required to relieve SOS genes from repression by either LexA [4] or a prophage repressor [41]. We conducted RNASeq analysis on both *recA* and *umuDAb* mutant strains of ADP1 and 17978 to test whether *recA* regulated these transcriptomes, and whether *umuDAb* was a global regulator of DNA damage-induced (and/or repressed) genes in a LexA-analogous manner.

**Figure 2:**
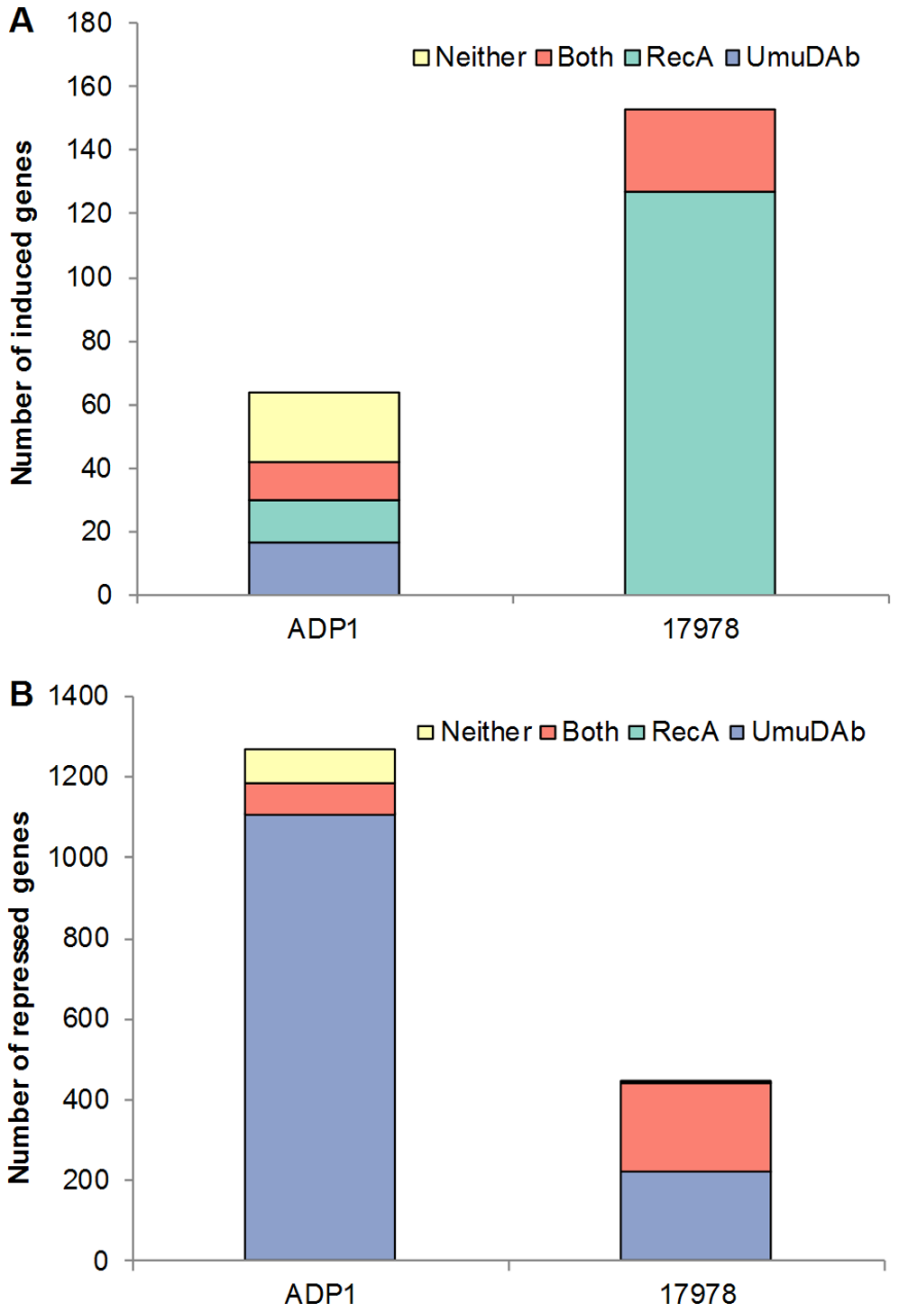
Distribution of regulation mechanisms for mitomycin C-induced and repressed transcriptome in ADP1 and 17978. The absolute number of genes induced (A) or repressed (panel B) by MMC in the transcriptome of ADP1 and 17978 is shown. The designation of regulon is represented by the following terms: Neither (genes requiring neither *umuDAb* nor *recA* for regulation), Both (genes requiring both *umuDAb* and *recA* for regulation), RecA (genes requiring only *recA* for regulation), or UmuDAb (genes requiring only *umuDAb* for regulation). (A) Many more repressed genes were observed in ADP1 than 17978, with UmuDAb sufficing for this repression in most genes; 17978 repressed genes required either UmuDAb or both UmuDAb and RecA. (B) A greater number of induced genes was observed in 17978 than ADP1, and these genes required either RecA or both RecA and UmuDAb. In comparison, ADP1 induced genes belong to four regulons (Neither, Both, RecA, or UmuDAb).

These experiments demonstrated a complex picture of regulation, with the ADP1 transcriptome possessing four regulons of induced genes that differentially required *umuDAb* and *recA* (Table 1). Figure 2A shows these four regulons, which were supported by statistical testing (repeated measures analysis of variance within each regulon; p<0.05). Twelve genes were regulated by both *umuDAb* and *recA*; 13 genes required *recA* only. Unexpectedly, we found a regulon of 22 genes that were induced after DNA damage but required neither *recA* nor *umuDAb* for this induction. Additionally, 17 genes were regulated only by *umuDAb*, but all of these were still moderately induced in the *umuDAb* mutant, having an average induction ratio of 1.70-fold (only slightly below the cutoff for being considered induced), and were not investigated further. These categories were validated by RT-qPCR experiments: *ddrR* required both *recA* and *umuDAb*, *dnaN* required only *recA*, and *ssb* and *nrdA* required neither *recA* nor *umuDAb*. In the eight induced operons, the regulation was the same throughout the operon, supporting the categorization and physiological relevance of the regulation method.

**Figure 3:**
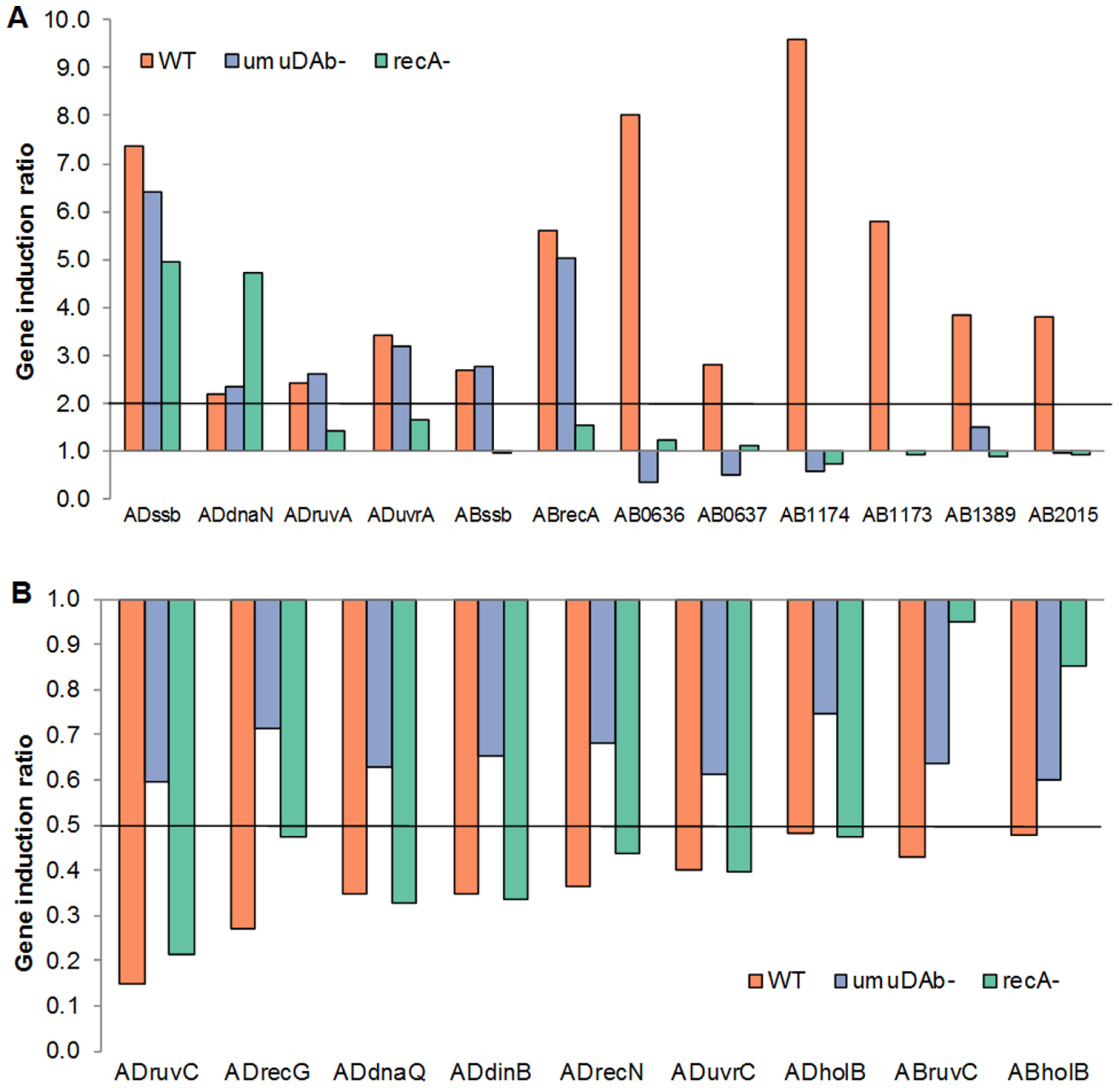
Mitomycin-C induced and repressed SOS genes in ADP1 and 17978 differentially require RecA and UmuDAb. Gene induction ratios obtained from RNASeq transcriptome experiments are shown, with the induction (panel A) and repression (panel B) of canonical SOS genes. Gene names prefaced by “AD” indicate ADP1 genes; “AB” indicates 17978 genes, with A1S numbers of 17978 genes listed for *umuDC* alleles. The placement of the horizontal axis in each panel represents the cutoff level for a gene to be considered induced (A) or repressed (B). Bars above the horizontal axis indicate induced genes (panel A), and bars below the horizontal axis indicate repressed genes (panel B), with bars rising either below (panel A) or above (panel B) this axis to have lost their induction or repression, respectively, in the *umuDAb* and/or *recA* mutant strains. (A) Induced genes did not require *umuDAb* in either ADP1 or 17978, except for the category of *umuDC* alleles, and *recA* was required for all induced 17978 genes but only some induced ADP1 genes (*ruvA* and *uvrA*). (B) Repressed genes required only *umuDAb* in ADP1 but required both *umuDAb* and *recA* in 17978.

This variety in regulatory requirements also extended to the induced SOS genes (Figure 3A). None of the five canonical SOS genes that were induced (*recA*, *dnaN, uvrA, ssb*, and *ruvA*) depended upon *umuDAb*. Only three of the five SOS genes were *recA*-regulated, none of the five were *umuDAb*-regulated, and strikingly, *ssb* was regulated by neither *recA* nor *umuDAb* (Figure 3A). This regulation was confirmed in RT-qPCR experiments.

In contrast, throughout the ADP1 genome, including all repressed SOS genes, 87% of the repressed genes required only *umuDAb* to be repressed, with just 6% requiring both *umuDAb* and *recA*, and 7% requiring neither of these genes for repression (Figure 2B).

### The *A. baumannii* DNA damage transcriptome requires RecA regulation and displays a specialized regulatory role for the UmuDAb repressor

In contrast to ADP1, 17978 exhibited only a *recA*-dependent path of inducing genes—with the exception of *recA* itself and A1S_2020, which were induced 2.0 to 2.2 –fold, respectively, in the *recA* mutant. However, the 17978 induced transcriptome contained two DNA-damage induced regulons: i) 123 genes regulated by *recA* (i.e. *umuDAb*-independent), and ii) 27 genes regulated by *recA* and *umuDAb* (i.e. *umuDAb*-dependent) (Table 3, Figure 2A). Within the *umuDAb*-independent regulon, there was a significant difference between the induction of the wild type *vs.* the *umuDAb* samples (p<0.05 in a Wilcoxon matched-pairs signed-ranks test), suggesting a possibly different role of *umuDAb* from simple repression. Consistent with the proportions of genes in these two regulons, 85% of the induced genes in the three prophages CP5, CP9, and CP14 required *recA* only, and this regulation was not significantly different for conserved hypothetical genes *vs.* genes typically found in bacteriophages (p>0.05, Fisher's exact test.). These observations were consistent with the possibility of gene repression by a prophage-encoded repressor. Of the 8 induced canonical SOS genes (which includes 6 alleles of *umuDC*), only the *umuDC* alleles were dependent on *umuDAb* for induction (Figure 3A). The *recA* and *ssb* genes' induction were *umuDAb* independent (Figure 3A). This regulation of *ssb*, *umuDAb*, and *recA* was confirmed in RT-qPCR experiments.

In the DNA damage-repressed transcriptome, this pattern was reversed: *umuDAb* was required for 99% of the genes' repression, with *recA* also required in ∼49% of the cases. This was also observed in the repressed SOS genes, where *umuDAb* was required for repression after DNA damage of *holB* and *ruvC*, but *recA* was required for repression as well (Figure 3B). However, repression of 7 of the 8 genes located on the plasmids pAB1 and pAB2 required both *umuDAb* and *recA*.

**Figure 4:**
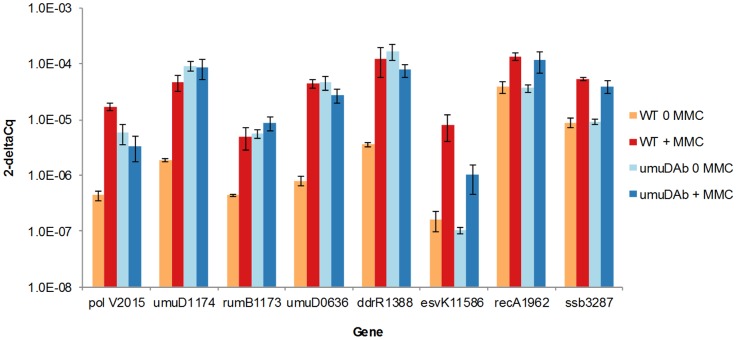
RT-qPCR experiments indicate that *umuDAb* is required for repression of error-prone polymerase components, not all DNA damage-induced genes. Delta Cq values from RT-qPCR experiments measuring expression of selected *A. baumannii* ATCC 17978 genes demonstrates the repressing activity of UmuDAb only for error prone polymerase components. The expression of each gene in both wild type and *umuDAb* null mutant is shown, with gene identity and A1S number listed on the x axis. Each gene was assayed in one RT-qPCR experiment (plate), with error bars indicating standard error of the mean from technical triplicates of biological triplicates.

We further tested whether all of the prophage-encoded error-prone polymerase alleles (CP5 (A1S_1173/1174, *umuDrumB*) and CP9 (A1S_2014-15)) were regulated similarly to their chromosomal counterparts *umuDC* (A1S_0636-0637) and the regulatory *umuDAb* gene (A1S_1389). All were regulated by *recA* and de-repressed in the *umuDAb* mutant (i.e. had high expression in the absence of MMC exposure), with this regulation confirmed by RT-qPCR experiments (Figure 4). This was not observed for non- *umuDC*-related alleles, either prophage- or chromosomally- encoded (*recA*, *esvK1*, and *ssb*): although *recA*-dependent, they were not regulated by *umuDAb* or de-repressed in the *umuDAb* mutant (Figure 4).

### DNA damage in *A. baumannii* induces bacteriophage particle production that contain virulence genes

PHAST characterization of the prophages apparently encoded by the three induced regions of the 17978 chromosome indicated that the majority of each prophage's genes (65% in CP14, 70% in CP5, and 81% in CP9) were either conserved hypothetical phage genes or phage genes of a function identified by homology (Figure 1B, Table 4). Of these genes typically found in bacteriophages, 65 - 78% (depending on the CP) were most similar to phage genes from the viral family *Siphoviridae*. In CP9, 68% of the phage genes were most similar (identity ranging from 60 – 100%) to genes in the *Acinetobacter* siphovirus BΦ-B1251, which was found in a sewage sample and lysed a carbapenem-resistant *A. baumannii* clinical isolate [42]. Both CP5 and CP14 were composed of a variety of phages' genes, with no one species being in the majority. This difference was statistically significant (p<0.05, Fisher's exact test).

**Table 4:** Description of gene functions in order of appearance in each prophage in 17978.

Phage Region	A1S	Name/Function	Category
**CP5**	1140	ribonuclease	DNA Metabolism/Replication
	1141	global regulatory protein	Lysogeny/Regulation
	1142	aspartate kinase	Moron
	3603	hypothetical protein	Phage hypothetical protein
	3604	hypothetical protein	Phage hypothetical protein
	3605	hypothetical protein	Phage hypothetical protein
	3606	hypothetical protein	Phage hypothetical protein
	1143	hypothetical protein	Hypothetical protein
	1144	repressor	Lysogeny/Regulation
	1145	putative Cro protein	Lysogeny/Regulation
	3607	Rha family transcriptional regulator	Lysogeny/Regulation
	3608	hypothetical protein	Phage hypothetical protein
	1146	methytransferase	DNA Metabolism/Replication
	1147	site-specific DNA methylase-like protein	DNA Metabolism/Replication
	3609	hypothetical protein	Phage hypothetical protein
	3610	hypothetical protein	Hypothetical protein
	3611	Hypothetical protein	Phage hypothetical protein
	3612	HNH endonuclease	DNA Metabolism/Replication
	3613	hypothetical protein	Phage hypothetical protein
	1148	hypothetical protein	Phage hypothetical protein
	3614	hypothetical protein	Phage hypothetical protein
	1149	hypothetical protein	Phage hypothetical protein
	1150	hypothetical protein	Phage hypothetical protein
	1151	phage protein	Phage hypothetical protein
	1152	terminase, large subunit	Capsid/DNA packaging
	1153	portal protein	Capsid/DNA packaging
	1154	phage head morphogenesis protein	Capsid/DNA packaging
	3615	hypothetical protein	Hypothetical protein
	3616	hypothetical protein	Hypothetical protein
	1155	putative head protein	Capsid/DNA packaging
	1156	hypothetical protein	Phage hypothetical protein
	1157	major capsid protein	Capsid/DNA packaging
	1158	putative signal peptide	Moron
	1159	hypothetical protein	Phage hypothetical protein
	3617	hypothetical protein	Hypothetical protein
	1160	hypothetical protein	Hypothetical protein
	1161	hypothetical protein	Hypothetical protein
	1162	hypothetical protein	Hypothetical protein
	3618	hypothetical protein	Hypothetical protein
	1163	hypothetical protein	Hypothetical protein
	3619	hypothetical protein	Hypothetical protein
	1164	tail tape measure protein	Tail Morphogenesis
	1165	tail tape measure protein	Tail Morphogenesis
	1166	hypothetical protein	Phage hypothetical protein
	1167	tail assembly structural protein	Tail Morphogenesis
	1168	hypothetical protein	Phage hypothetical protein
	3620	hypothetical protein	Phage hypothetical protein
	1169	putative tail protein	Tail Morphogenesis
	1170	putative tail protein	Tail Morphogenesis
	3621	hypothetical protein	Phage hypothetical protein
	1171	hypothetical protein	Phage hypothetical protein
	1172	IS903 transposase	Transposase/Insertion sequence
	1173	*rumB*, error-prone legion bypass DNA polymerase V	Error-prone polymerase/Moron
	1174	*umuD*	Error-prone polymerase/Moron
	3622	hypothetical protein	Phage hypothetical protein
	1175	phage integrase	Lysogeny/Regulation
**CP9**	2040	putative integrase	Lysogeny/Regulation
	3779	hypothetical protein	Hypothetical protein
	3778	hypothetical protein	Phage hypothetical protein
	3777	hypothetical protein	Phage hypothetical protein
	3776	hypothetical protein	Phage hypothetical protein
	2039	putative phage nucleotide-binding protein	DNA Metabolism/Replication
	2038	hypothetical protein	Hypothetical protein
	3775	hypothetical protein	Hypothetical protein
	3774	hypothetical protein	Hypothetical protein
	2037	*esvI/*putative repressor cI	Lysogeny/Regulation
	3773	hypothetical protein	Hypothetical protein
	3772	hypothetical protein	Phage hypothetical protein
	2036	DNA cytosine methyltransferase	DNA Metabolism/Replication
	3771	hypothetical protein	Phage hypothetical protein
	3770	hypothetical protein	Hypothetical protein
	2035	HNH nuclease	DNA Metabolism/Replication
	3769	hypothetical protein	Hypothetical protein
	3768	hypothetical protein	Hypothetical protein
	2034	putative antirepressor	Lysogeny/Regulation
	2033	hypothetical protein	Phage hypothetical protein
	2032	hypothetical protein	Phage hypothetical protein
	2031	phage protein	Phage hypothetical protein
	2030	hypothetical protein	Phage hypothetical protein
	2029	hypothetical protein	Phage hypothetical protein
	2028	phage head morphogenesis protein	Capsid/DNA Packaging
	3767	hypothetical protein	Phage hypothetical protein
	3766	hypothetical protein	Phage hypothetical protein
	2027	hypothetical protein	Phage hypothetical protein
	2026	major capsid protein	Capsid/DNA Packaging
	2025	major capsid protein	Capsid/DNA Packaging
	3765	hypothetical protein	Phage hypothetical protein
	3764	hypothetical protein	Phage hypothetical protein
	2024	hypothetical protein	Phage hypothetical protein
	3763	hypothetical protein	Phage hypothetical protein
	2023	hypothetical protein	Phage hypothetical protein
	3762	hypothetical protein	Phage hypothetical protein
	3761	hypothetical protein	Phage hypothetical protein
	2022	putative tail fiber	Tail Morphogenesis
	2021	hypothetical protein	Phage hypothetical protein
	3760	hypothetical protein	Phage hypothetical protein
	2020	hypothetical protein	Phage hypothetical protein
	2019	hypothetical protein	Phage hypothetical protein
	3759	putative lipoprotein	Phage hypothetical protein
	2018	tail tape measure protein	Tail Morphogenesis
	3758	hypothetical protein	Phage hypothetical protein
	3756	hypothetical protein	Hypothetical protein
	3757	hypothetical protein	Phage hypothetical protein
	2017	tail fiber	Tail Morphogenesis
	3755	holin	Lysis
	2016	lysozyme	Lysis
	3754	hypothetical protein	Phage hypothetical protein
	2015	*umuC,* error-prone DNA polymerase	Error-prone polymerase
	2014	hypothetical protein	Phage hypothetical protein
**CP14**	1580	integrase	Lysogeny/Regulation
	3683	hypothetical protein	Hypothetical protein
	3684	hypothetical protein	Hypothetical protein
	1581	putative methyltransferase	DNA Metabolism/Replication
	3685	hypothetical protein	Hypothetical protein
	3686	repressor	Lysogeny/Regulation
	3687	hypothetical protein	Hypothetical protein
	1582	transcriptional regulator Cro/Cl family	Lysogeny/Regulation
	1583	hypothetical protein	Hypothetical protein
	1584	hypothetical protein	Phage hypothetical protein
	1585	putative replicative DNA helicase	DNA Metabolism/Replication
	3688	hypothetical protein	Hypothetical protein
	3689	hypothetical protein	Hypothetical protein
	3690	hypothetical protein	Hypothetical protein
	3691	hypothetical protein	Hypothetical protein
	3692	hypothetical protein	Hypothetical protein
	3693	hypothetical protein	Phage hypothetical protein
	3694	hypothetical protein	Phage hypothetical protein
	3695	hypothetical protein	Hypothetical protein
	3696	hypothetical protein	Hypothetical protein
	1586	*esvK1/*ethanol-stimulated virulence protein	DNA Metabolism/Replication
	3697	HNH nuclease	DNA Metabolism/Replication
	3698	hypothetical protein	Hypothetical protein
	1587	*esvK2/*terminase	Capsid/DNA packaging
	1588	large terminase	Capsid/DNA packaging
	1589	portal protein	Capsid/DNA packaging
	1590	Pro-head protease	Capsid/DNA packaging
	1591	putative head major capsid protein	Capsid/DNA packaging
	3699	hypothetical protein	Hypothetical protein
	1592	head/tail adapter protein	Capsid/DNA packaging
	1593	hypothetical protein	Phage hypothetical protein
	1594	hypothetical protein	Phage hypothetical protein
	1595	major tail subunit	Tail Morphogenesis
	3700	hypothetical protein	Hypothetical protein
	3701	putative lipoprotein	Phage hypothetical protein
	1596	tail length tape measure protein	Tail Morphogenesis
	1597	tail tape measure protein	Tail Morphogenesis
	3702	hypothetical protein	Phage hypothetical protein
	1598	putative tail fiber protein	Tail Morphogenesis
	1599	hypothetical protein	Phage hypothetical protein
	3703	hypothetical protein	Phage hypothetical protein
	3704	hypothetical protein	Lysis
	1600	lysozyme	Lysis

All three prophage regions were within the size range for non-*Bacillus* siphovirus genomes (14–56 kb [43], [44]) and were organized into modules of (in this order): lysogeny/regulation, DNA metabolism, DNA packaging and head, tail, and lysis genes (Figure 1B), which is the same organization as in genomes from the family *Siphoviridae* [43], [45]. This analysis and annotation by the PHAST software, as well as manual characterization and genome size of the three prophage regions, suggested that the 45 kb CP5 was an intact prophage, encoding the requisite morphological (capsid, packaging, tail), DNA replication and lysogeny regulation (including repressors; Table 4) gene products indicative of a functional prophage. However, the 49 kb CP9 and the 22 kb CP14 also contained these genes and may be intact prophages as well. Thus the composition as well as the induction of these prophages differed from the (uninduced) prophage loci present in ADP1, the larger (∼53 kb) of which contains only roughly one third of its genes as phage genes (either as conserved hypotheticals or of known function) (Figure 1A), [28], most of which resembled genes from the Family *Myoviridae*.

**Figure 5:**
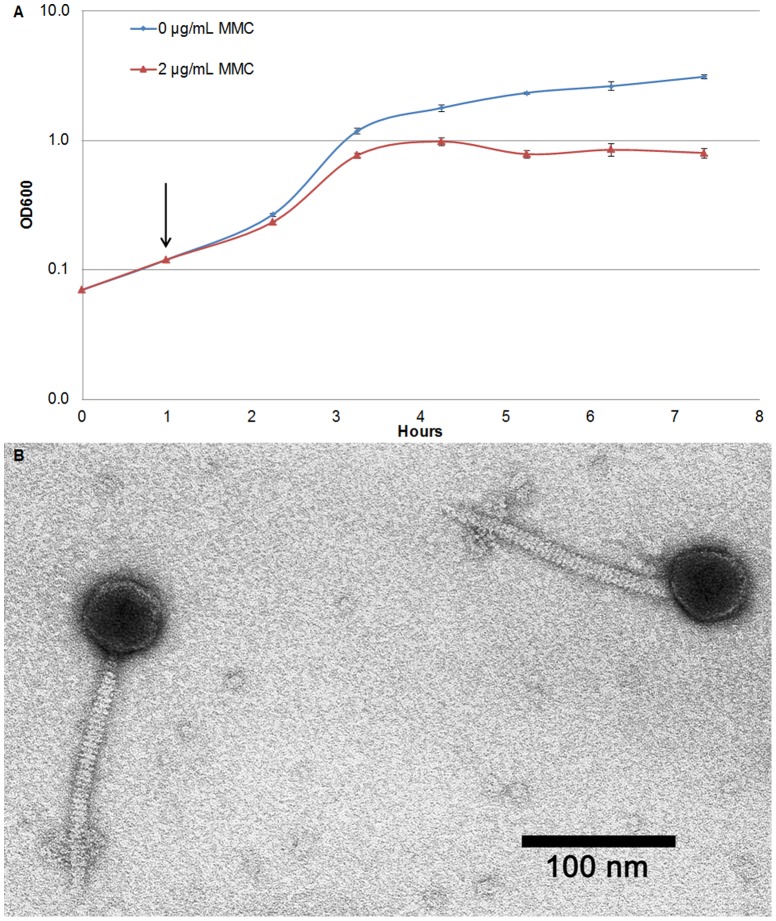
Mitomycin C treatment induces production of bacteriophage particles in 17978. (A) Overnight LB cultures of 17978 cells were diluted into fresh LB medium and grown for 0.75 hours before addition of 2 μg/mL MMC. After approximately two hours of MMC treatment, the optical density leveled off and decreased slightly but continued to increase in the absence of MMC treatment. Error bars represent standard error of the mean from three independent experiments. (B) Electron micrograph of bacteriophage particles at 100,000× magnification, showing polyhedral capsid, long, flexible tail and tail fibers. Results shown are representative of three independent experiments producing and imaging bacteriophage particles.

We hypothesized that because ∼99% of all the genes in these prophages were induced after DNA damage, bacteriophage particles might be produced under these conditions. When 17978 cells were grown in LB medium in the presence of MMC, a decrease in culture turbidity was observed beginning around two hours post-exposure, relative to untreated cells (Figure 5A). Transmission electron microscopy was used to visualize intact phage particles of uniform morphology from filtered supernatants of these cultures in three independent experiments. Morphological analyses of these phages showed them to have a non-enveloped capsid of approximately 57 nm in diameter, and a long, thin (11 nm), flexible tail of approximately 167 nm that possessed tail fibers (Figure 5B). These morphological features, together with the size, content and organization of the three prophage regions, suggest that the phage particles may belong to the viral family *Siphoviridae*. Bacteriophages in the *Myoviridae* family visually resemble siphoviruses but possess a wider and inflexible tail. Furthermore, viruses in the *Myoviridae* family are lytic, and thus not consistent with the temperate nature of the phages that we observed.

We tested whether these similar-looking bacteriophages represented the products of CP5, CP9, CP14, or a mixture of all three of these prophages, as was suggested by the induction of genes in all three prophage regions. Phage particles were purified away from bacterial fragments and chromosomal DNA in both MMC-treated and untreated cultures by DNAse treatment of supernatant that had been 0.22 μm filtered and precipitated. PCR amplification experiments were performed on these DNAse-treated, purified samples to determine whether genes from each prophage region were present in the particles we observed. Primers that amplified portions of *rumB* (from CP5), *esvI* and *umuC* A1S_2015 (from CP9), and *esvK1* (from CP14) all yielded PCR products only from 3 hour-MMC-treated, purified culture supernatants, but not from untreated, purified culture supernatants, in three independent experiments. This suggests that all three putative prophage regions might produce phage particles when induced by MMC, although it was not unambiguously determined that these particles are encoded by each of the three prophage regions independently, or whether one of these phage, e.g. CP5, might have served as a helper virus for the production of particles containing CP9 or CP14 prophage DNA.

We next tested the hypothesis that the *umuD-rumB* (A1S_1173-1174) operon that we observed in the phage lysate is responsible for the DNA damage-induced mutagenesis previously observed in this strain [14], [32]. Compared to the frequency of rifampin-resistant mutants observed in wild type 17978 cells after UV exposure, a *rumB* null mutant displayed only approximately ∼40% of the rifampin mutation frequency after DNA damage (in four independent experiments). This suggests that if CP5 produced phage particles, these could transduce these genes into a new host and so allow error-prone replication of DNA in this host. However, a similar, partial (∼65%) reduction of rifampin resistance frequency in a non-phage encoded *umuD* (A1S_0636) null mutant was also observed (in six independent experiments). The apparent redundancy of these error-prone polymerases in the DNA damage-inducible mutagenesis occurring in the 17978 strain is likely a reflection of these polymerases being of bacteriophage as well as bacterial origin in this species.

## Discussion

These transcriptome studies of *A. baumannii* ATCC 17978 and *A. baylyi* ADP1 indicated that a genome-wide system of inducing and repressing genes after DNA damage exists in these species. Between 2% and 4% of these species' genes were induced after mitomycin C treatment, but their distribution throughout the chromosome differed greatly, with localization of most (∼90%) of the 17978 genes into three prophages but wide dispersal of ADP1 induced genes throughout the chromosome. There was little overlap in the DNA damage-induced transcriptomes of these organisms with either canonical SOS genes (only 11–17% of which were induced) or each other (only *recA, ssb, umuDAb, ddrR*, and *gst* were induced in both species). *recA*, *ssb*, and *umuD* are core SOS genes, whereas *gst* encodes a member of the glutathione *S*-transferase (GST) family, which protects against oxidative stress and detoxifies endogenous, xenobiotic and antimicrobial compounds [35]. *A. baumannii* ATCC 17978, like many proteobacteria, possesses multiple (9) *gst* genes [46], as does *A. baylyi* ADP1 [28]. The induced *gst* genes (A1S_0408 and ACIAD0445) share 69% amino acid identity, and are present in a highly syntenic chromosomal location in these two species, allowing for the possibility that A1S_0408 and ACIAD0445 may be members of the GST family that participate in the DNA damage response.

Besides a subset of the canonical SOS genes, stress proteins and chaperones, ADP1 induced the genes of a CRISPR/Cas system, which are bacterial adaptive immunity/defense modules. The cell processes foreign, e.g. bacteriophage, DNA molecules and forms a CRISPR array locus in the chromosome composed of short segments of these DNA sequences [47]. The next time similar DNA molecules enter the cell, Cascade proteins (*cas* gene-encoded, typically adjacent to the CRISPR repeat locus) and transcribed CRISPR sequences bind to and cleave the incoming foreign DNA. The *A. baylyi* ADP1 Type I-F CRISPR/Cas locus consists of the Cascade proteins encoded by *cas3/cas2* (ACIAD2477), as well as two conserved hypothetical genes, *csy2*, *csy3*, *cas6*, and *cas1* (ACIAD2479-2484, which were induced by MMC). It is intriguing that in *A. baylyi* ADP1 cells, which are naturally competent for the uptake of and transformation with DNA [15], this CRISPR/Cas defense against foreign DNA appears to be functional. Short transcribed CRISPR RNA molecules (crRNA), identical to those comprising the CRISPR repeats adjacent to the induced ACIAD2479-2484 genes, accumulate in ADP1 cells after treatment with nalidixic acid [48], a well-known inducer of the SOS response. The dependence of these crRNA molecules' formation on new protein synthesis [48] is consistent with induction of the *cas* genes that we observed. A link between a CRISPR/Cas system and DNA repair has been observed in *E. coli*, where the Cas1 nuclease YgbT acts on both branched DNAs and in antiviral immunity [49]. However, to our knowledge, this is the first evidence of transcriptional induction of a CRISPR/Cas system gene by DNA damage. To the limited extent that CRISPR/Cas genes' expression has been studied, a constitutive level has been assumed [48], but the uninduced level of A1S_2479-2484 expression is modest, being below the average uninduced level of the 66 induced genes, but approximately four times the detection threshold of the RNASeq experiments.

Our data are largely consistent with those observed in recent microarray studies of *A. baumannii* ATCC 17978 in which 39 genes were induced more than 1.5-fold after MMC treatment [19], with 77% of that study's genes also induced in our experiments. The greater number of induced genes observed in our study (152), as well as the variation in the specific identity of the induced genes may be because of the different methodologies used (RNA-Seq *vs* microarray), and also because Aranda *et al*. used a rich medium source (LB broth), a shorter induction time of two hours, and one-quarter the amount of MMC as in this study. The invariant conservation of the induction of all error-prone polymerases and polymerase components in this and other studies [19], [32], however, supports the centrality of these genes to the DNA damage response of this species.

Further transcriptional profiling of *umuDAb* and *recA* mutant strains of both species after MMC treatment allowed determination of the roles of these putative regulators in the DNA damage responses. In the DNA damage-induced transcriptomes, *recA* was required for the induction of only 38% of the ADP1 induced genes, but virtually all of the 17978 induced genes, which is consistent with both the known SOS response mechanism [4] and the involvement of *recA* in antimicrobial resistance, general stress responses, and virulence in 17978 [23]. This *recA* dependence is also consistent with the repression of the prophage genes by a prophage-encoded repressor [41] as opposed to a LexA-like, UmuDAb-mediated repression of these genes. However, we observed that 9–19% of each of the three prophage genomes required *umuDAb* for gene induction in addition to *recA*, which argues against a solitary action of RecA-facilitated autocleavage of a prophage repressor in the response we observed. The action of UmuDAb, a potential LexA homolog, was complex in both species, playing a role in only 44% of ADP1 induced genes, and in 16% of 17978 induced genes, including both those encoded in prophages and in the chromosome. The large number of repressed genes in the DNA damage transcriptomes, especially of ADP1 (Figure 2B), was unexpected, with the repressor action of UmuDAb being consistent with its involvement in the repression of the vast majority of these genes in ADP1, although its action may be indirect rather than direct.

The de-repression that we observed of *ddrR* and all *umuDC* alleles in a null *umuDAb* mutant is consistent with recent observations that UmuDAb binds to, and regulates, the promoters of these genes in *A. baumannii* ATCC 17978 [19], although those studies used a *umuDAb* insertion mutant and not a null mutant. However, our genome-wide profiling of *umuDAb* regulation of induced genes found that unlike for the *umuDC* alleles, the induction of the majority (83.5%) of all genes in 17978 was *umuDAb*-independent. Either UmuDAb is not the sole LexA-like repressor in this species, or has a mechanism of action unlike LexA, because a LexA-regulated regulon of DNA damage-induced genes would have become de-repressed in the absence of DNA damage, which was not observed (except for the *umuDC* and *ddrR* genes). Furthermore, *umuDAb* was required for the induction of genes that are not error-prone polymerases and which were encoded in prophage regions (Table 3). These data suggest that UmuDAb does not serve as a direct replacement of LexA for the entire DNA damage regulon in this genus, instead serving a more specialized role in repressing error-prone polymerases. This specialized UmuDAb role invokes an additional DNA damage-related repressor to regulate gene expression after DNA damage, which is consistent with the failure of RecA to regulate its own induction, seen both in this study and previously for *A. baylyi* ADP1 [16] and *A. baumannii* [32].

In having multiple *umuDAb*-dependent and –independent regulons, the behavior of *Acinetobacter* in regulating their genes after DNA damage is more like its closer pseudomonad relatives, which contain multiple regulons of DNA damage-induced genes involving different (LexA) repressor proteins [12], than it is to enteric bacteria such as *E. coli*. These *Acinetobacter* species, like *P. aeruginosa*, also repressed many more genes than they induced in response to DNA damage, and both genera repressed multiple canonical SOS genes in a *lexA*-independent manner (*recG* in ADP1, and *holB* and *ruvC* in both ADP1 and 17978), and induced *nrdAB* and prophage genes [34].

Our observation of the 17978 strain possessing DNA damage-inducible bacteriophages that encode mutation-inducing (error prone) polymerase genes may hold significant implications for the evolution of virulence and antibiotic resistance in related strains. CP5 encodes the *umuDrumB* operon, which this study found to be responsible for at least half of the DNA damage-induced mutagenesis, while CP9 encodes A1S_2015, annotated as an “error-prone lesion bypass DNA polymerase V” that might also contribute to mutagenesis after DNA damage [32]. Multiple DNA damage-inducing agents–UV-C exposure [14], [32] as well as methyl methanesulfonate, dessication, and ciprofloxacin [32]–are capable of inducing mutagenesis (as measured by rifampin resistance) in *A. baumannii* ATCC 17978 and AB0057 [14]. *A. baumannii* strains AB0057 and 3909 also contain CP5 that encodes the *umuDrumB* genes [38], while *A. baumannii* ATCC 19606 and D1279779, strains not investigated by DiNocera *et al.*, also possess a very similar CP5-like prophage region that encodes *umuDrumB* (Figure S1). This indicates the possibility of a widespread mechanism in this species for spread of these error-prone polymerase genes in response to multiple stimuli. Virulence-associated genes such as *esvK1* and *esvK2* (encoded in CP14), and *esvI* (encoded in CP9) that contributed to ethanol-stimulated virulence in a model of *C. elegans* infection by the 17978 strain [27] are also encoded by these prophages and could contribute to the evolution of strains through transduction by bacteriophages that may be produced from, or encapsidate, the genomes of CP5, CP9, or CP14, although these phages have not yet been shown to infect other hosts.

The overall patterns of UmuDAb and RecA usage in these species suggests that diverse mechanisms exist in *A. baylyi* ADP1 for the repression and induction of genes, which include a regulon induced by neither UmuDAb nor RecA. In contrast, *A. baumannii* ATCC 17978 almost universally depends on RecA (as well as UmuDAb) but also uses additional, unknown repressors and/or regulators, possibly of prophage origin, in addition to UmuDAb. These species therefore offer robust model systems in which to study the processes of gene regulation after DNA damage, with *A. baumannii* additionally posing a relevant biological problem in its possible dissemination of error-prone polymerases.

## Supporting Information

Figure S1 **CP5-like prophage regions present in** ***A. baumannii*** **strains.** The three to-scale diagrams indicate CP-like prophage regions present in *A. baumannii* strains ATCC 19606 and D1279779. Analysis and image production was performed using the PHAST webserver, with the color-coding indicating the likely function assigned to each coding sequence. The numbered bar indicates the nucleotide number in the genome, with coding regions in the three forward frames shown above the bar and coding regions in the three reverse frames shown below the bar for each strain.
(TIF)

Table S1 **PCR primers used in constructing** ***umuDAb*** **,** ***umuD*** **, and** ***rumB*** **mutants of** ***A. baumannii*** **ATCC 17978.**
(DOCX)

Table S2 **Primers used in RT-qPCR experiments in** ***A. baylyi*** **ADP1.**
(DOCX)

Table S3 **Primers used in RT-qPCR experiments in** ***A. baumannii*** **ATCC 17978.**
(DOCX)
