# Supplementary figures and images for: Prophage Induction and Differential RecA and UmuDAb Transcriptome Regulation in the DNA Damage Responses of Acinetobacter baumannii and Acinetobacter baylyi
Source: PLoS One. 2014 Apr 7;9(4):e93861. doi: 10.1371/journal.pone.0093861 (PMC3978071; doi:10.1371/journal.pone.0093861)

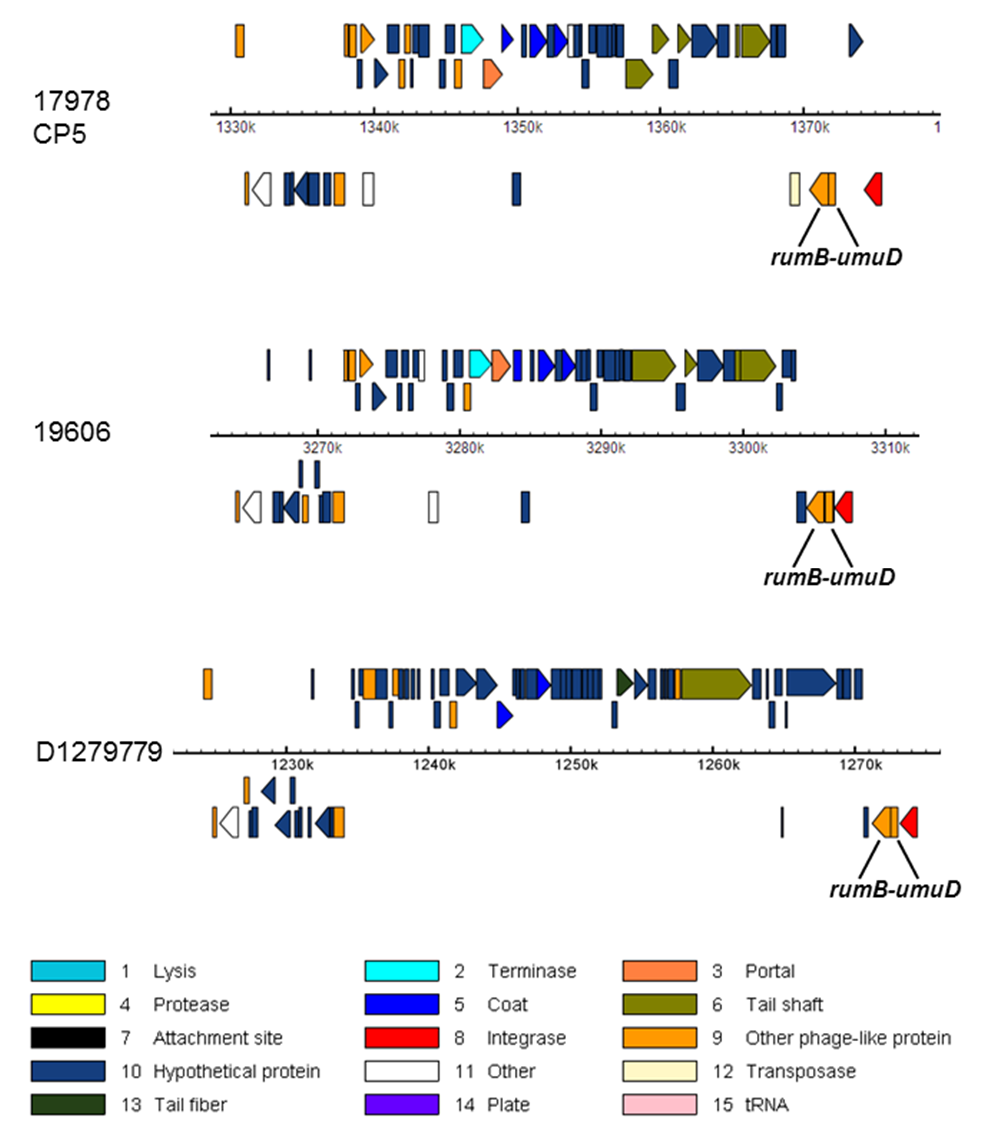

Supplement: Figure S1 — CP5-like prophage regions present in A. baumannii strains. The three to-scale diagrams indicate CP-like prophage regions present in A. baumannii strains ATCC 19606 and D1279779. Analysis and image production was performed using the PHAST webserver, with the color-coding indicating the likely function assigned to each coding sequence. The numbered bar indicates the nucleotide number in the genome, with coding regions in the three forward frames shown above the bar and coding regions in the three reverse frames shown below the bar for each strain. (TIF) [file pone.0093861.s001.tif]
