# Supplementary material for: Prophage Induction and Differential RecA and UmuDAb Transcriptome Regulation in the DNA Damage Responses of Acinetobacter baumannii and Acinetobacter baylyi
Source: PLoS One. 2014 Apr 7;9(4):e93861. doi: 10.1371/journal.pone.0093861 (PMC3978071; doi:10.1371/journal.pone.0093861)
Supplement: Table S1 — PCR primers used in constructing umuDAb , umuD , and rumB mutants of A. baumannii ATCC 17978. (DOCX) [file pone.0093861.s002.docx]

Table S1. PCR primers used in constructing *umuDAb*, *umuD*, and *rumB* mutants of *A. bauamannii* ATCC 17978

| Primer name | Primer sequence | Reference |
| --- | --- | --- |
| upUDAbFor | ATGCTTATGGACGCTTTACG | This study |
| upintUDAbRev | CCCAGCTGGCAATTCCGGTCACCGTTCAAATCCTACAC | This study |
| dwUDAbRev | CCATTTCCGCAGGTATTAAG | This study |
| dwintUDAbFor | CTTGACGAGTTCTTCTGAACCTATAACCTCAAAC | This study |
| upUmuDFor | GCAGCAGCACATTTTGAGC | This study |
| upintUmuDRev | CCCAGCTGGCAATTCCGGATTAAAACCTCTTGA | This study |
| dwUmuDRev | TAACCTTAAATCATCAGTCGG | This study |
| dwintUmuDFor | CTTGACGAGTTCTTCTGAAGTTAGAAAAAGATCATGAATC | This study |
| upRumBFor | CTATTGCCATCTCCAGTGC | This study |
| upintRumBSS | AAGCGTGCATAATAAGCCCTCATGCATGCACAAGAAGCAT | This study |
| dwRumBRev | TCCTAATGGAACGTCTTCAT | This study |
| dwintRumBSS | CGGCAACCTTGGGCAGCGACTTAATGCCGTGTCATTTT | This study |
| StrepSpecFor | GCTGCCCAAGGTTGCCG | This study |
| StrepSpecRev | AGGGCTTATTATGCACGCTT | This study |
| Kmup | CCGGAATTGCCAGCTGGG | [24] |
| Kmdw | TTCAGAAGAACTCGTCAAG | [24] |
