# Supplementary material for: Prophage Induction and Differential RecA and UmuDAb Transcriptome Regulation in the DNA Damage Responses of Acinetobacter baumannii and Acinetobacter baylyi
Source: PLoS One. 2014 Apr 7;9(4):e93861. doi: 10.1371/journal.pone.0093861 (PMC3978071; doi:10.1371/journal.pone.0093861)
Supplement: Table S2 — Primers used in RT-qPCR experiments in A. baylyi ADP1. (DOCX) [file pone.0093861.s003.docx]

| **Name** | **Sequence** |
| --- | --- |
| ADPssbRTFor | CAGACCGAATGGCACCGTAT |
| ADPssbRTRev | AACGCTCCTGACCATTCTGG |
| ADP006RTFor | CAGGCGAACTGTTTTGGTCAT |
| ADP006RTRev | AGCCTTACACAAGCACAGCAC |
| ADP2481RTFor | CCGTTTTACGCCTGCTCAAC |
| ADP2481RTRev | ATGGCAAGATGCGGTTGGTT |
| ADP2482RTFor | ATGGTCGTTGGTTATGGCGT |
| ADP2482RTRev | TCCTGTTCAGATGGTTCGGC |
| ADP0724RTFor | ATGACCGTCGTCGTACTCAC |
| ADP0724RTRev | GCTGTGCAAATTCTTCGCCA |
| 16SrRNA#RTFor | CCACACTGGGACTGAGACAC |
| 16SrRNA#2RTRev | AACCAGGTAAGCCTCCTCCT |
| ddrR#RTFor | ATACCGAACAAGCCGAGCAT |
| ddrR#2RTRev | AGGCATGACTAAAGCCAGCA |
| umuDAb#RTFor | GGAGCATGTCGAGCAGAGTC |
| umuDAb#2RTRev | TCACCTGCTTTGGCCGTAAT |
| dnaN#RTFor | TGCCAATAACCCAGAGCAGG |
| dnaN#2RTRev | AACCGACTGATTGGCCTCAG |
| RecA#RTFor | AAGGTGAAATGGGCGACTCC |
| RecA#2RTRev | CTGGGCTGCCAAACATTACG |

Table S2. Primers used in RT-qPCR experiments in *A. baylyi* ADP1
