# Supplementary material for: Prophage Induction and Differential RecA and UmuDAb Transcriptome Regulation in the DNA Damage Responses of Acinetobacter baumannii and Acinetobacter baylyi
Source: PLoS One. 2014 Apr 7;9(4):e93861. doi: 10.1371/journal.pone.0093861 (PMC3978071; doi:10.1371/journal.pone.0093861)
Supplement: Table S3 — Primers used in RT-qPCR experiments in A. baumannii ATCC 17978. (DOCX) [file pone.0093861.s004.docx]

Table S3. Primers used in RT-qPCR experiments in *A. baumannii* ATCC 17978

| **Name** | **Sequence** |
| --- | --- |
| Abau0636RTFor | AAGTTGTTGCTCGGTCTCGG |
| Abau0636RTRev | TGCCGGTGATGGAAATCCTG |
| AbauddrRRTFor | GGAAAGTGAAGCAGCCGAAAG |
| AbauddrRRTRev | TGGGTAAGGGGATGTAAGCCT |
| Abau2015RTFor | TTTGCACACTCTAGCCCGTT |
| Abau2015RTRev | TCAAGCGCAGTCAAAACCAC |
| Abau1173RTFor | CGTTTCTGAAGTTTGGGGCG |
| Abau1173RTRev | CGTAGATCGTGCGAGCCATA |
| 17ssb#2RTFor | CAGATGCTGGTTGTTGTGGC |
| 17ssb#2RTRev | ATAACAACCAAGGTGGGGGC |
| 17978umuDAbFor | TACCACATTCCTTTGGCGAC |
| 17978umuDAbRev | TCCGGCATCTAACATGGACA |
| 17978RecARTFor | CCACTGGTGGTAACGCTCTT |
| 17978RecARTRev | ACGGAGGAGCCATTTTGTTC |
| 1797816rRNARTFor | GGGAGAAAGCAGGGGATCTT |
| 1797816rRNARTRev | CGGATCATCCTCTCAGACCC |
| 17978esvIRTFor | GAGTGGCTCCCTTTACCTGA |
| 17978esvIRTRev | ATCGCCAGATTGCATGTCAC |
| 17978esvK1RTFor | CAATTGGGGTTCTGGTCGTG |
| 17978esvK1RTRev | ATCTGTTCCACCTCTTGCCA |
| 17978umuDCRTFor | CCGTGTTTGGCATCAAGACT |
| 17978umuDCRTRev | CTCTCGACATGAACGAGCAC |
| 17978esvIRTFor | GAGTGGCTCCCTTTACCTGA |
| 17978esvIRTRev | ATCGCCAGATTGCATGTCAC |
|  |  |
